# Impact of aortic valve calcification volume on left ventricular systolic function in patients with severe aortic stenosis undergoing transcatheter aortic valve implantation^☆^

**DOI:** 10.1016/j.ahjo.2025.100562

**Published:** 2025-06-03

**Authors:** Helen S. Anwar, Magdy Algowhary, Mohamed Aboel-Kassem F. Abdelmegid, Hatem A. Helmy, J.M. Montero-Cabezas, Frank Van Der Kley

**Affiliations:** a Department of Cardiovascular Medicine, Assiut University Heart Hospital, Assiut University, Assiut, Egypt; b Department of Cardiology, Leiden University Medical Center, Leiden, the Netherlands

**Keywords:** Aortic valve calcification, Left ventricular systolic function, Severe aortic stenosis, Transcatheter aortic valve implantation

## Abstract

**Background:**

Aortic valve calcification (AVC) has been linked to negative cardiac outcomes in patients with aortic stenosis (AS). Given the limited understanding of its specific contributions, we aimed to investigate the interaction between AVC and the left ventricular (LV) systolic function in patients with severe AS and LV systolic dysfunction who underwent transcatheter aortic valve implantation (TAVI).

**Materials and methods:**

An observational study of 75 patients with severe AS and LV ejection fraction (EF) ≤ 50 % who underwent TAVI. AVC volume was determined by ECG-gated contrast-enhanced multidetector computed tomography (MDCT) using specific software (3Mensio Structural Heart version 10.4, Pie Medical Imaging, Maastricht, the Netherlands). Patients were categorized into two groups based on LV systolic function recovery 30 days after TAVI defined by ≥10 % absolute increase in LVEF compared to baseline.

**Results:**

AVC volume showed a statistically significant negative correlation with the baseline LVEF (*r* = −0.33, *P* = 0.008) and a statistically significant positive correlation with the percentage of change in LVEF as compared to the baseline (*r* = 0.38, *P* = 0.001). In the logistic regression for post-TAVI LV systolic function recovery, AVC volume was associated with an increased likelihood of LV systolic function recovery.

**Conclusion:**

AVC volume has a paradoxical association with LV systolic function. Patients with a higher AVC volume had a more depressed baseline LV systolic function and a greater likelihood of LV systolic function recovery after TAVI.

## Highlights

• AVC is one of the independent predictors of mortality in aortic stenosis patients.
• AVC is associated with negative outcomes post-TAVI.
• Higher AVC is associated with reduced baseline LVEF with likelihood of LVEF recovery after TAVI.
• AVC quantification is a valuable prognostic tool predicting patients likely to experience LVEF recovery post-TAVI.

## 1 Introduction

Aortic stenosis (AS) is the most prevalent valvular heart disease in Europe and North America, affecting approximately 3.4 % of elderly populations [1]. Being a disease of the left ventricle (LV), studies have shown that nearly one-third of patients with severe AS experience LV systolic dysfunction due to chronic pressure overload. This chronic pressure overload leads to myocardial hypertrophy and, eventually, fibrosis [2,3]. Notably, this LV dysfunction can be reversible in up to two-thirds of patients following therapeutic interventions, particularly transcatheter aortic valve implantation (TAVI) [3,4]. The mechanism underlying the recovery of LV systolic function post-intervention is not yet fully understood. Various factors may influence it, all of which warrant further investigation to improve patient outcomes.

Aortic valve calcification (AVC) is the hallmark of degenerative AS and serves as one of the independent predictors of mortality following AS diagnosis [5]. In patients with severe AS undergoing TAVI, AVC plays a critical role in ensuring the stable anchoring of the prosthesis to the aortic annulus, particularly with the use of self-expanding valves, However, AVC is also associated with an increased risk of TAVI-related complications, such as conduction disturbances, paravalvular leakage, and annulus rupture [6]. Therefore, AVC measurement should be considered not only for diagnostic purposes but also as a key factor in risk stratification for patients with AS.

The potential impact of AVC on LV systolic function in patients with severe AS at baseline and following TAVI is not well understood and remains underexplored. Therefore, this study aimed to investigate the association between AVC and baseline LV systolic function and its effect on LV systolic function recovery in patients with severe AS and LV ejection fraction (EF) ≤ 50 % undergoing TAVI.

## 2 Methods

### 2.1 Patient population and data collection

**Fig. 1:**
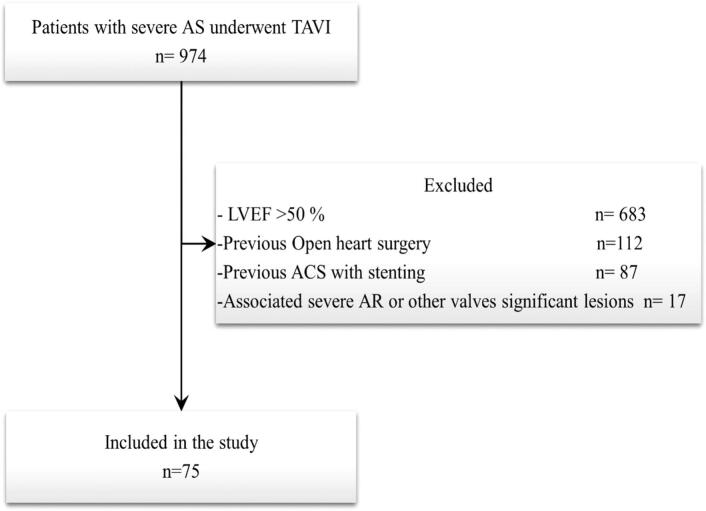
Flow chart of study population:

An observational retrospective study in which 974 patients with severe AS underwent TAVI between January 2019 and December 2023 at the Leiden University Medical Center, Leiden, the Netherlands were reviewed. We included only patients with reduced LV systolic function (20 % ≤ LVEF ≤50 %). Patients with previous open-heart surgery (coronary artery bypass or valve replacement), previous percutaneous coronary intervention due to ST-elevation myocardial infarction or non-ST-elevation myocardial infarction, those with concomitant severe mitral or aortic valve regurgitation and significant post TAVI paravalvular leakage were excluded to minimize the confounding factors that could independently affect LV systolic function. This rigorous selection yielded a study population of 75 patients (Fig. 1). All patients had transthoracic echocardiogram (TTE), ECG-gated contrast-enhanced multidetector computed tomography (MDCT), and coronary angiography before TAVI. A TTE 30 days after TAVI, was conducted as part of a routine follow-up. The departmental electronic medical record (EPD-vision 12.14.0.1; Leiden University Medical Center, Leiden, the Netherlands) was used to collect all demographic and clinical data. The Scientific Institutional Review Board approved this retrospective analysis.

### 2.2 TTE data acquisition and measurements

TTE was conducted using Vivid-7, E9, or E95 systems (General Electric Vingmed, Horten, Norway). Offline echocardiographic analysis was performed with EchoPac software (EchoPac version 204; GE Medical Systems, Horten, Norway). Based on the Bernoulli equation, peak and mean transaortic gradients were calculated using continuous-wave Doppler imaging of apical 3- or 5-chamber views. Aortic valve area (AVA) was estimated via the continuity equation and normalized to body surface area (indexed AVA). Severe AS was defined as an AVA <1.0 cm^2^ or indexed AVA below 0.6 cm^2^/m^2^, combined with a mean transaortic pressure gradient of at least 40 mmHg or a peak transaortic jet velocity of 4 m/s or higher [7].

Stroke volume (SV) and stroke volume index (SV index) were determined using the velocity-time integral, measured via pulsed-wave Doppler recordings of the left ventricular outflow tract (LVOT). These recordings were acquired from the apical 3- or 5-chamber view, with the sample volume positioned just below the AV. Based on these parameters, severe AS can be categorized hemodynamically as follows:

• High gradient AS, characterized by AVA <1.0 cm^2^ and a mean pressure gradient of at least 40 mmHg, irrespective of SV index value.
• Low-flow, low-gradient (LFLG) AS, defined by AVA < 1.0 cm^2^, a mean pressure gradient below 40 mmHg and an SV index ≤35 mL/m^2^ [8].

LV end-diastolic and end-systolic volumes were measured using planimetry in the apical 2- and 4-chamber views and the LVEF was calculated via Simpson's biplane method. LV dimensions, including intraventricular septal thickness, LV end-diastolic diameter, and posterior wall thickness, were assessed in the parasternal long-axis view during end-diastole. LV mass was calculated using the Devereux formula and adjusted to the body surface area (LVMI) [9].

Aortic, mitral, and tricuspid valve regurgitation severity were identified and graded according to current recommendations [10].

### 2.3 MDCT acquisition and analysis

Contrast-enhanced specific TAVI protocol was acquired allowing anatomical assessment of the aortic valve, aortic root and arch, and the aorto-ilio-femoral vasculature using a 320 MDCT scanner (Aquilion ONE, Toshiba Medical Systems, Otawara, Japan). Prospective ECG gated data acquisition for the thorax (the heart and aortic root) with a slice thickness of 0.5 mm then non-ECG gated acquisition for the abdomen and pelvis with a slice thickness of 2.0 mm. Image reconstruction was done at different percentages of the cardiac cycle (R-R interval) [11]. Data processing and analysis were performed in a remote workstation with dedicated software (3Mensio Structural Heart version 10.4, Pie Medical Imaging, Maastricht, the Netherlands).

**Fig. 2:**
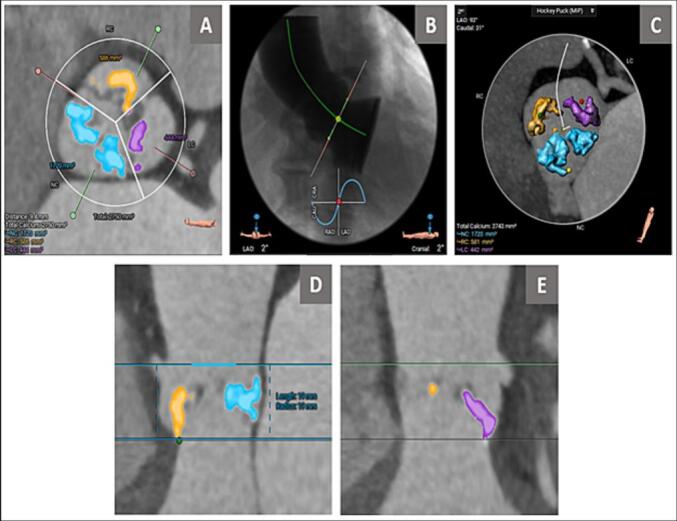
Example of one of our patients, Aortic valve calcification volume evaluation using 3Mensio CT software:

For the evaluation of AVC volume, as previously recommended, an individual threshold of the Hounsfield units (HU) was used to distinguish between calcium and contrast medium which was calculated automatically by the 3Mensio CT software according to the contrast enhancement of the cardiac structure and in LVOT with minimal manual adjustment [12]. The mean of HU was 548.5 ± 100 which matched with the threshold used by other studies conducted by Everts et al. [13] and Ludwig et al. [14]. Firstly, the aortic annulus was identified as the standard method (the most basal attachment point of each aortic valve cusps) [15]. Then, the calcium quantification feature in the 3Mensio CT software will be used, with manual adjustment of the region of interest. AVC was defined as calcification within the aortic valve leaflets, aortic annulus, or aortic wall below the coronary ostium with the exclusion of calcification of the coronary arteries and in the LVOT [16]. AVC was expressed as a volume in mm^3^, (Fig. 2).

### 2.4 Coronary angiography

Coronary angiography was done as part of routine institutional work-up for all TAVI candidate patients before the procedure. Percutaneous coronary intervention was performed in patients with significant coronary artery disease (defined by the presence of coronary stenosis >70 % in a major epicardial coronary artery) [8].

### 2.5 TAVI procedure

The decision regarding the access route, valve type, and size was made by the local heart team based on the pre-TAVI MDCT data analysis and patient characteristics. The TAVI procedure was performed according to standard practice using local anesthesia and either trans-femoral or trans-axillary vascular arterial access when required. Balloon expandable (Edwards Lifesciences SAPIEN3/3 Ultra) or self-expandable (Medtronic Evolut PRO/PRO+ or Boston Scientific Accurate Neo2) valves were used.

### 2.6 30 days follow-up TTE

A TTE was done for all patients 30 days after TAVI using the same protocol previously mentioned in the pre-TAVI evaluation.

LV systolic function recovery was defined by ≥10 % absolute increase in LVEF as compared to the baseline [17]. Accordingly, patients were categorized into two distinct groups: recovered or non-recovered LV systolic function.

## 3 Statistical analysis

Shapiro-Wilk test was used to test the normality of data. Continuous data are presented as mean ± standard deviation (SD) if normally distributed and as median (interquartile range) (IQR) if not normally distributed. Categorical data are presented as absolute numbers and percentages. Paired *t*-test was used to compare the variables before and after TAVI. The independent t-test and Mann-Whitney test were used to compare continuous variables between groups while the chi-square test was used to compare categorical variables. The Spearman correlation tests were used to identify the correlation between variables and AVC volume. Uni-and multivariate binary logistic regression models were done for possible predictors of LV systolic function recovery.

All statistical analyses were performed with SPSS software version 29.0 (IBM, Armonk, NY). Significant variables in the univariate logistic regression with *p*-value <0.05 were entered in a multivariate logistic regression analysis to obtain the independent predictors of LV function recovery (odds ratio (OR), 95 % confidence interval (CI)). A 2-tailed p-value <0.05 is considered statistically significant.

## 4 Results

### 4.1 Baseline patient characteristics

Seventy-five patients with severe AS and LV dysfunction (20 % ≤ LVEF ≤50 %) met the inclusion criteria. Baseline characteristics and echocardiographic data of the study population are presented in Table 1. The mean age of the cohort was 82.8 ± 8.6 years. Male patients comprised 57.3 % of the study population. Forty-one patients were in sinus rhythm, twenty-one had AF, and seven had permanent pacemakers. The mean of transaortic mean pressure gradient was 42.6 ± 13.3 mmHg, while the mean AVA was 0.76 ± 0.20 cm^2^. 52.0 % of patients had high-gradient AS. The mean baseline LVEF was 35.5 ± 6.7 %. The mean LVMI was 144.0 ± 36.9 g/m^2^. 74.7 % of our study population had a high surgical risk with logistic Euro SCORE *I* ≥ 10 %.

**Table 1:** Baseline, laboratory, and TTE data of enrolled patients:

Variables	*N* = 75
Age (years) ± SD	82.8 ± 8.6
Male sex, n (%)	43 (57.3 %)
Body mass index (kg/m2), ±SD	26.5 ± 5.3
Body surface area (m2), ±SD	1.88 ± 0.22
Hypertension, n (%)	49 (65.3 %)
Diabetes mellitus, n (%)	21 (28.0 %)
CAD	
No CAD, n (%)	58 (77.4 %)
One vessel, n (%)	10 (13.3 %)
Two vessels, n (%)	7(9.3 %)
ECG	
Sinus rhythm, n (%)	41(45.7 %)
AF, n (%)	27(36.0 %)
Pacemaker, n (%)	7(9.3 %)
Pre TAVI LBBB, n (%)	23 (30.7 %)
Laboratory data	
Hemoglobin (mmol/l) ± SD	7.72 ± 0.94
Estimated Glomerular filtration rate (ml/min//1.73m^2^) ± SD	56.13 ± 19.87
Logistic Euro SCORE I %	
Low surgical risk <10 %, n (%)	19 (25.3 %)
High surgical risk ≥10 %, n (%)	59 (74.7 %)
Mean transaortic pressure gradient (mmHg) ± SD	42.6 ± 13.3
Transaortic maximum velocity (m/s) ± SD	3.9 ± 0.6
Aortic valve area (cm) ± SD	0.76 ± 0.20
Indexed Aortic valve area (cm/m^2^) ± SD	0.39 ± 0.10
Stroke volume index (ml/m^2^) ± SD	33.7 ± 6.5
AS type	
High gradient, n (%)	39 (52.0 %)
LFLG with reduced EF, n (%)	36 (48.0 %)
LVEF (%) ± SD	35.5 ± 6.7
21–30 %, n (%)	16 (21.4 %)
31–40 %, n (%)	42 (56.0 %)
41–50 %, n (%)	17 (22.6 %)
LAVI (ml/m^2^) ± SD	48.9 ± 12.2
LVMI (g/m^2^) ± SD	144.0 ± 36.9
AR grade	
No, n (%)	37 (49.3 %)
Mild, n (%)	26 (34.7 %)
Moderate, n (%)	12 (16.0 %)
MR grade	
Mild, n (%)	36 (48.0 %)
Moderate, n (%)	32 (42.6 %)
Moderately severe, n (%)	7 (9.4 %)
TR grade	
Mild, n (%)	48 (64.0 %)
Moderate, n (%)	23 (30.7 %)
Moderately severe, n (%)	4 (5.3 %)
PASP (mmHg) ± SD	38.0 ± 10.0
TAPSE (mm) ± SD	18.8 ± 2.6

Data was expressed in the form of mean ± SD, and frequency (percentage).

**AF:** atrial fibrillation, **AR**: aortic regurgitation, **AS**: aortic stenosis, **CAD**: coronary artery disease, **ECG**: electrocardiogram, **IVSD**: interventricular septum diastolic diameter, **LAVI**: left atrial volume index, **LBBB**: left bundle branch block, **LFLG**: low flow low gradient, **LVEF**: left ventricular ejection fraction, **LVMI**: left ventricular mass index, **MR**: mitral regurgitation, **PASP**: pulmonary artery systolic pressure, **TAPSE**: tricuspid annular plane systolic excursion, **TR**: tricuspid regurgitation.

### 4.2 Pre-TAVI MDCT workup and procedural data of the enrolled patients

86.7 % of enrolled patients had a tricuspid aortic valve while 13.3 % had a bicuspid aortic valve. The median (IQR) AVC volume was 960 (500–1573) mm^3^. Regarding vascular access, all our patients underwent transfemoral TAVI either single (57.4 %) or two groins (42.6 %). 72.0 % of our patients received Edwards Lifesciences SAPIEN3 / 3 Ultra valve while in 26.7 % of patients, Medtronic Evolut PRO/ PRO+ was used and only one patient had a Boston Scientific Accurate Neo 2 valve (1.3 %) (Table 2).

**Table 2:** Pre-TAVI MDCT workup and procedural data:

Variables	N = 75
Type of native aortic valve	
Tricuspid n (%)	65 (86.7 %)
Bicuspid n (%)	10 (13.3 %)
Maximum annulus diameter (mm) ± SD	28.0 ± 3.0
Minimum annulus diameter (mm) ± SD	22.0 ± 2.34
Perimeter (mm) ± SD	79.8 ± 8.2
Area (mm^2^) ± SD	488.2 ± 90.5
SOV (mm) ± SD	35.4 ± 4.4
STJ (mm) ± SD	28.6 ± 4.2
Ascending Aorta (mm) ± SD	32.9 ± 5.3
STJ height (mm) ± SD	23.4 ± 3.6
Femoral access site	
Right common femoral artery diameter (mm) ± SD	8.3 ± 1.6
Tortuosity	
Mild (angel >60 degree) n (%)	61 (81.3 %)
Moderate (30 < angel<60 degree) n (%)	12 (16.0 %)
Severe (angel<30 degree) n (%)	2 (2.75)
Calcification	
No/Dots n (%)	32 (42.7 %)
Mild (< 90 degree of artery circumference) n (%)	26 (34.7 %)
Moderate (90–270 degree) n (%)	12 (16.0 %)
Severe (270–360 degree) n (%)	5 (6.6 %)
AVC volume(mm^3^)	960
(median, IQR)	(500–1573)
Hounsfield units (HU) ± SD	548.5 ± 100
Vascular access	
Transfemoral single groin	43 (57.4 %)
Transfemoral two groins	32 (42.6 %)
Type of TAVI valve	
SAPIEN 3 / 3 ultra n (%)	45 (72.0 %)
Evolut pro / pro+ n (%)	20 (26.7 %)
Accurate Neo 2 n (%)	1 (1.3 %)

Data was expressed in the form of mean ± SD, median (IQR), and frequency (percentage).

**AVC:** Aortic valve calcification, **SOV:** sinus of Valsalva, **STJ:** Sinotubular junction.

### 4.3 Comparison between recovered and non-recovered groups post TAVI

46 (61.3 %) patients showed a ≥ 10 % absolute increase in LVEF in a 30-day TTE follow-up compared to the baseline. Accordingly, patients were categorized into recovered (46 patients, 61.3 %) and non-recovered groups (29 patients, 38.7 %). Table 3 shows baseline demographic, MDCT, and echocardiographic differences between the two groups. Baseline characteristics were similar between the two groups apart from most patients in the recovered group having baseline sinus rhythm compared to the non-recovered group (65.2 % vs 37.9 %, *P* = 0.04). Regarding the echocardiographic findings, patients in the recovered group had lower baseline LVEF (34.0 ± 5.6 vs 38.9 ± 7.7 %), higher transaortic mean pressure gradient (46.3 ± 12.8 mmHg vs 36.6 ± 11.8 mmHg), smaller AVA (0.7 ± 0.1 cm vs 0.8 ± 0.2 cm) and higher LVMI (153.4 ± 38.7 g/m^2^ vs 129.3 ± 28.6 g/m^2^) than the non-recovered group (*P* = 0.001, < 0.001, < 0.001, 0.003, respectively). Patients with LVEF 31–40 % showed the highest percentage of recovery among other LVEF categories (*P* = 0.04). AVC volume was significantly higher in the recovered than non-recovered group (*P* = 0.02).

**Table 3:** Comparison of data between patients with recovered and non-recovered LV systolic function regarding demographic, TTE, and MDCT workup data:

Variables	Recovered (*N* = 46)	Non-recovered (*N* = 29)	*P* value
Age (years) ± SD	83.5 ± 8.6	81.9 ± 8.9	0.45
Male sex, n (%)	24 (52.2 %)	19 (65.5 %)	0.34
Body mass index (kg/m2) ± SD	26.1 ± 5.5	27.2 ± 5.1	0.35
Hypertension, n (%)	30 (65.2 %)	19(65.5 %)	0.9
Diabetes mellitus, n (%)	13 (28.3 %)	8 (27.6 %)	0.9
CAD			
No CAD, n (%)	33 (71.7 %)	25 (86.2 %)	0.08
One vessel, n (%)	6 (13.1 %)	4 (13.8 %)	
Two vessels, n (%)	7 (15.2 %)	0 (0.0 %)	
ECG			
Sinus rhythm, n (%)	30 (65.2 %)	11 (37.9 %)	0.04
AF, n (%)	12 (26.1 %)	15 (51.8 %)	
Pacemaker, n (%)	4 (8.7 %)	3 (10.3 %)	
Pre TAVI LBBB, n (%)	14 (30.4 %)	9 (31.0 %)	0.90
Laboratory data			
Hemoglobin (mmol/l) ± SD	7.7 ± 0.8	7.7 ± 1.2	0.92
Estimated Glomerular filtration rate (ml/min//1.73m^2^) ± SD	58.5 ± 19.7	52.4 ± 19.9	0.20
PG mean (mmHg) ± SD	46.3 ± 12.8	36.6 ± 11.8	<0.001
AVA (cm) ± SD	0.7 ± 0.1	0.8 ± 0.2	<0.001
Indexed AVA (cm/m^2^) ± SD	0.3 ± 0.1	0.4 ± 0.1	0.002
AS type			
High gradient	29(63.0 %)	10(34.5 %)	0.02
LFLG with reduced EF	17(37.0 %)	19 (65.5 %)	
Pre TAVI LVEF (%) ± SD	34.0 ± 5.6	38.9 ± 7.7	0.001
Pre-TAVI LVEF grading (%)			
21–30 %, n (%)	11 (32.9 %)	5 (17.2 %)	0.04
31–40 %, n (%)	29 (63.0 %)	13 (44.8 %)	
41–50 %, n (%)	6 (13.0 %)	11 (37.9 %)	
Pre-TAVI LVMI (g/m^2^) ± SD	153.4 ± 38.7	129.3 ± 28.6	0.003
AVC volume(mm^3^) (median, IQR)	1004 (657–1800)	750 (275–1301)	0.02
Type of native aortic valve			
Tricuspid, n (%)	40 (87.0 %)	25 (86.2 %)	0.65
Bicuspid, n (%)	6 (13.0 %)	4 (13.8 %)	
Type of TAVI valve			
SAPIEN 3/3 ultra, n (%)	30(65.2 %)	24 (82.8 %)	0.22
Evolut pro/pro+, n (%)	15 (32.6 %)	5(17.7 %)	
Accurate Neo 2, n (%)	1 (2.2 %)	0 (0.0 %)	
Final PVL			
Mild and less, n (%)	44 (95.7 %)	28 (96.6 %)	0.9
Moderate, n (%)	2(4.3 %)	1 (3.4 %)	
Post TAVI PPM, n (%)	2 (4.3 %)	2(6.9 %)	0.6

Data was expressed in the form of mean ± SD, median (IQR), and frequency (percentage). P-value is significant if <0.05.

**AF:** atrial fibrillation, **AS**: aortic stenosis, **AVA**: aortic valve area, **AVC**: Aortic valve calcification, **CAD**: coronary artery disease, **ECG**: electrocardiogram. **LBBB**: left bundle branch block, **LVEF;** left ventricular ejection fraction, **LFLG**: low flow low gradient, **LVMI**: left ventricular mass index, **PG mean**: Transaortic mean pressure gradient, **PPM:** permanent pacemaker, **PVL:** para valvular leakage.

**Fig. 3:**
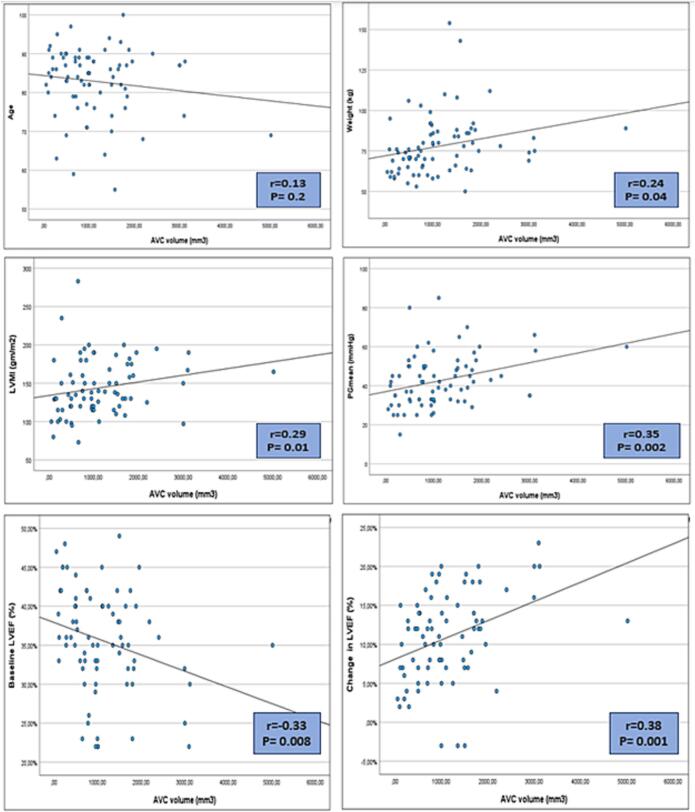
Correlation between AVC volume and baseline and echocardiographic-derived parameters of patients:

With focusing on the AVC, Fig. 3, a statistically significant positive correlation was detected between AVC and patient weight (*r* = 0.24, P = 0.04). Considering the echocardiographic parameters, our study showed a statistically significant positive correlation between AVC volume and transaortic PG mean and LVMI (*r* = 0.35, 0.29, *P* = 0.002,0.01 respectively). Regarding LVEF, our results showed a statistically significant negative correlation between AVC volume and baseline LVEF (*r* = −0.33, *P* = 0.008), while a statistically significant positive correlation with the percentage of change in LVEF compared to the baseline (*r* = 0.38 *P* = 0.001).

AVC volume was higher in males than females and in patients with high gradient AS than LFLG AS (*P* < 0.001, = 0.04, respectively) (Table 4).

**Table 4:** Association of AVC volume and patients baseline characteristics:

Variables	AVC volume (median, IQR)	P value
Sex		
Male	1350 (700–1800)	**<0.001**
Female	765 (322–975)	

HTN		
Yes	970 (510–1387)	0.7
No	956 (499–1660)	

DM		
Yes	956 (570–1539)	0.7
No	970 (500–1653)	

CAD		
No CAD	970 (510–1543)	0.9
One vessel	960 (375–1718)	
Two vessels	792 (700–1800)	

Rhythm		
Sinus	979 (690–1681)	0.1
AF	950 (264–1500)	

AS type		
High gradient	1107 (660–800)	0.04
LFLG	950 (339–1217)	

Data was expressed in the form of median (IQR). P value is significant if <0.05.

**AF**: atrial fibrillation, **AS:** aortic stenosis, **AVC:** Aortic valve calcification, **CAD:** coronary artery disease, **DM:** diabetes mellitus, **HTN:** hypertension, **LFLG**: low flow low gradient, **TAVI**: transcatheter aortic valve implantation.

Investigating the factors associated with early LV systolic function recovery, the univariable logistic regression analysis indicated that patients with sinus rhythm, a higher transaortic mean gradient, a smaller AVA, a higher LVMI, a lower baseline LVEF, and a greater AVC volume had a higher likelihood of LV systolic function recovery. However, in the multivariate analysis, only having a smaller AVA (OR 0.03, 95 % CI: 0.00–1.13, *P* = 0.02), and a higher LVMI (OR 1.04, 95 % CI: 1.00–1.08, P = 0.02) were independently associated with an increased probability of early LV systolic function recovery after TAVI, Table 5.

**Table 5:** Univariate and multivariate analysis of the predictors of recovery of LV systolic function post-TAVI (≥ 10 % absolute increase in LVEF compared to baseline):

Variables	Univariate analysis				B	Multivariate analysis		
	B	Odds'ratio	95 % confidence interval	P value		Odds' ratio	95 % confidence interval	P value
Age	0.03	1.0	0.97–1.07	0.45	–	–	–	**–**
Female sex	0.55	1.7	0.67–4.54	0.25	–	–	–	**–**
Diabetes	0.03	1.1	0.36–2.91	0.94	–	–	–	**–**
Hypertension	−0.01	0.98	0.37–2.62	0.97	–	–	–	**–**
BMI	0.01	1.00	0.91–1.11	0.85	–	–	–	**–**
Hgb (mmol/l)	1.06	1.11	0.65–1.87	0.69	–	–	–	**–**
Sinus rhythm	1.4	4.02	1.49–10.79	**0.006**	1.2	3.35	0.75–14.99	0.11
Pre-TAVI LBBB	−0.03	0.97	0.35–2.66	0.95	–	–	–	–
CAD	0.9	2.46	0.71–8.46	0.15	–	–	–	–
AVA (cm)	−4.13	0.16	0.001–0.25	**0.003**	−5.96	0.03	0.00–1.13	**0.02**
PG mean	0.07	1.07	1.02–1.12	**0.004**	–	–	–	–
LVMI (gm/m^2^)	0.02	1.02	1.00–1.04	**0.008**	0.04	1.04	1.00–1.08	**0.02**
LVEF (%)	−0.10	0.90	0.83–0.98	**0.01**	0.01	1.01	0.88–1.16	0.84
AVC volume (mm^3^)	0.001	1.00	1.00–1.01	**0.02**	0.001	1.00	1.00–1.00	0.09

**AVA:** aortic valve area, **AVC:** aortic valve calcification, **BMI**: body mass index, **CAD:** coronary artery disease, **Hgb:** hemoglobin, **LBBB:** left bundle branch block, **LVMI:** left ventricular mass index, **LVEF:** left ventricular ejection fraction, **PG mean:** transaortic mean pressure gradient.

## 5 Discussion

This study investigated the early LV systolic function recovery in a subgroup of patients with severe AS and reduced LV systolic function undergoing TAVI, focusing on the potential association between AVC volume and LV systolic function. The key findings of the present study were as follows [1]: Nearly two-thirds of our patients demonstrated early LV systolic function recovery after TAVI, with the effect being more pronounced in those with a baseline LVEF 31–40 %, (2) AVC burden differentiated between various haemodynamic subtypes of AS, (3) AVC volume had a negative impact on the LV systolic function in patients with severe AS, and (4). There was a positive association between AVC volume and LV systolic function recovery following TAVI.

Early LV systolic function recovery post-TAVI was noticed in 61.3 % of our patients, consistent with findings from a study done by Dauerman et al. [4]. This recovery rate is higher than that reported in the Placement of Aortic Transcatheter Valve (PARTNER) trials [18], where only around 50 % of patients exhibited early LVEF recovery. The higher recovery rate in our cohort may be attributed to different patient characteristics, as the PARTNER trial included patients with previous ST-elevation myocardial infarction, coronary artery bypass graft surgery, and associated severe mitral regurgitation which were exclusion criteria in our study.

The largest percentage of recovery following TAVI (61.5 % of recovered patients) was noted in those with a baseline LVEF (31–40 %) aligning with data from a previous study where they described that the largest increase in LVEF after TAVI occurred in patients with reduced baseline LVEF (33 ± 6 %) versus those with midrange baseline LVEF (45 ± 3 %) (*P* < 0.001) [19].

LV functional recovery occurs significantly in patients with higher baseline transaortic mean gradient, a finding consistent with the PARTNER trial and other studies [3,4,18]. Sinus rhythm and smaller AVA were also significantly more prevalent in the recovered group, corroborating observations made by Kolte et al. [20].

An intriguing finding of our study is that AVC volume was significantly higher in patients with recovered LV systolic function compared to those without recovery. Focusing on AVC, notably, male patients exhibited greater AVC volumes than female patients (P < 0.001), which is consistent with the findings by Singh et al. [21]. Previous studies have also identified associations between AVC and various cardiovascular risk factors including age, obesity, hypertension, and diabetes mellitus [22,23]. In alignment with these studies, a statistically significant positive correlation was detected between AVC volume and obesity measured by body weight. In contrast to these studies, no associations between AVC and age, hypertension, or diabetes were detected in our study. This discrepancy may be explained by the characteristics of our cohort, which consisted of elderly patients (age > 70 years old), adequately treated hypertensive patients, and a small number of diabetic patients.

Furthermore, our findings were consistent with earlier studies that demonstrated an association between AVC and the severity of AS [24,25]. Specifically, a positive correlation between AVC volume and transaortic mean pressure gradient was observed. There was a discrepancy in AVC volume in different hemodynamic types of AS, in which patients with high gradient AS had higher levels of calcification compared to those with LFLG AS. This finding aligns with the results reported by Evertz et al. [13].

Regarding LV systolic function, a paradoxical association was observed between AVC volume and LV systolic function. Specifically, AVC volume showed a negative correlation with baseline LVEF and a positive correlation with the percentage of change in LVEF following TAVI indicating a potential for LV systolic function recovery. The direct interaction between AVC and LV systolic function remains incompletely understood. However, emerging evidence suggests that AVC is an active pathological process involving the aortic valve, which may have a deleterious impact on the LV. This process is characterized by osteoblastic cell infiltration and the subsequent deposition of calcific material on the aortic valve [26]. This condition contributes to increased valve stiffness, reduced leaflet mobility, and progressive valve narrowing. The resulting disturbed flow from the calcific narrowed aortic valve creates downstream effects on the LV, primarily manifesting as LV hypertrophy due to the chronically increased afterload [27]. When the hypertrophic response surpasses a critical threshold, the myocardium may undergo apoptosis and be replaced by fibrous tissue leading to impaired LV systolic function [27,28]. Higher AVC is, the stronger, and the longer the period of afterload due to AS. TAVI alleviates this pressure afterload, thereby allowing for potential recovery of LV function.

Among the various factors influencing early LV systolic function recovery following TAVI, our findings indicated that patients with high AVC volume exhibited a greater tendency for LV systolic function recovery. However, high AVC volume did not emerge as an independent predictor of recovery. In contrast, a smaller AVA and a higher LVMI were identified as independent predictors of LV systolic function recovery according to our study.

Our study has strength points: Firstly, to our knowledge, our study is the first to assess the associations between AVC, baseline LV systolic function, and its recovery after TAVI in patients with AS and reduced LV systolic function. Secondly, we incorporate multiple exclusion criteria that could influence LV systolic function; the elimination of these confounders enhances the accuracy of our findings.

### 5.1 Study limitations

The present study has limitations. It was a retrospective single-center study. The relatively small sample size so the results will need to be investigated in a larger population to correctly evaluate the prediction model.

## 6 Conclusion

AVC is not merely a benign physiological consequence of aging, rather, it exerts a deleterious impact on cardiac structure and function. A paradoxical association between AVC volume and LVEF, where a higher AVC volume is negatively correlated with the baseline LVEF. Interestingly, patients with higher AVC volumes demonstrated a greater likelihood of LV systolic function recovery following TAVI.

## CRediT authorship contribution statement

**Helen S. Anwar:** Writing – original draft, Methodology, Conceptualization. **Magdy Algowhary:** Conceptualization. **Mohamed Aboel-Kassem F. Abdelmegid:** Writing – review & editing. **Hatem A. Helmy:** Writing – review & editing. **J.M. Montero-Cabezas:** Writing – review & editing. **Frank Van Der Kley:** Writing – review & editing.

## Funding

The present article received no external funding.

## Declaration of competing interest

The authors have no potential conflicts of interest to disclose.
